# Drift drives the evolution of chromosome number II: The impact of range size on genome evolution in Carnivora

**DOI:** 10.1093/jhered/esae025

**Published:** 2024-05-07

**Authors:** Michelle M Jonika, Kayla T Wilhoit, Maximos Chin, Abhimanyu Arekere, Heath Blackmon

**Affiliations:** Department of Biology, Texas A&M University, College Station, TX, United States; Interdisciplinary Program in Genetics and Genomics, Texas A&M University, College Station, TX, United States; Department of Biology, Texas A&M University, College Station, TX, United States; Department of Biology, Texas A&M University, College Station, TX, United States; Department of Biology, Texas A&M University, College Station, TX, United States; Department of Biomedical Engineering, University of Texas, Austin, TX, United States; Department of Biology, Texas A&M University, College Station, TX, United States; Interdisciplinary Program in Genetics and Genomics, Texas A&M University, College Station, TX, United States; Ecology and Evolutionary Biology Interdepartmental Program, Texas A&M University, College Station, TX, United States

**Keywords:** Carnivora, chromosomal evolution, genetic drift, karyotype, speciation

## Abstract

Chromosome number is a fundamental genomic trait that is often the first recorded characteristic of a genome. Across large clades, a common pattern emerges: many or even most lineages exhibit relative stasis, while a handful of lineages or species exhibit striking variation. Despite recent developments in comparative methods, most of this heterogeneity is still poorly understood. It is essential to understand why some lineages have rapid rates of chromosome number evolution, as it can impact a variety of other traits. Previous research suggests that biased female meiotic drive may shape rates of karyotype evolution in some mammals. However, Carnivora exhibits variation that this female meiotic drive model cannot explain. We hypothesize that variation in effective population size may underlie rate variation in Carnivora. To test this hypothesis, we estimated rates of fusions and fissions while accounting for range size, which we use as a proxy for effective population size. We reason fusions and fissions are deleterious or underdominant and that only in lineages with small range sizes will these changes be able to fix due to genetic drift. In this study, we find that the rates of fusions and fissions are elevated in taxa with small range sizes relative to those with large range sizes. Based on these findings, we conclude that 1) naturally occurring structural mutations that change chromosome number are underdominant or mildly deleterious, and 2) when population sizes are small, structural rearrangements may play an important role in speciation and reduction in gene flow among populations.

## Introduction

Chromosome number is a fundamental genomic trait that is commonly one of the first recorded characteristics of a genome (Goldsmith 1919; Painter 1921). When observing chromosome numbers in large clades, a familiar pattern can often be noted: many or even most lineages exhibit relative stasis, while a handful of lineages or species exhibit striking variation. Despite recent developments in comparative methods, the causes of this heterogeneity are still poorly understood. It is essential to understand why some lineages exhibit rapid rates of chromosome number evolution, as it can impact a variety of other traits. Changes in chromosome number can directly or indirectly impact the evolution of haplodiploidy, recombination rates, reproductive isolation, and gene transcription (Stebbins 1958; Rieseberg 2001; Lockstone et al. 2007; Williams et al. 2008; Sun et al. 2013; Blackmon et al. 2019).

Karyotypes are reshaped through several mechanisms. In this study, we use fusion and fission to describe a decrease or increase of one in haploid chromosome number. However, these fusions and fissions likely represent multiple processes at the molecular level. For example, a chromosome number decrease can occur through a Robertsonian translocation and subsequent loss of nonessential DNA (Garagna et al. 1995; Schubert and Lysak 2011), or less frequently, the chromosome number decrease could be a true fusion of two chromosomes at the telomeres followed by loss or inactivation of one of the centromeres (Gordon et al. 2011; Miga 2017). Chromosome number increases typically involve the fission of a chromosome in the centromere region, followed by the generation of new telomeric sequences. Less frequently, chromosome number increase may be due to the duplication of an entire chromosome (Moretti and Sabato 1984). Whole or partial genome duplications can also increase chromosome number but have not been documented in mammals and will not be discussed further in our work.

Previous research suggests that biased female meiotic drive may shape rates of karyotype evolution in mammals (Blackmon et al. 2019). Female mammals have asymmetric meiosis (i.e. the four products of meiosis will include three eliminated polar bodies and one that will become the oocyte). Asymmetric meiosis can allow the meiotic drive to occur, where some chromosomes are more likely to segregate into the egg versus the polar body. The meiotic drive can lead to the rapid fixation of mutations (Rhoades 1942; Sandler and Novitski 1957). Female meiotic drive where bibrachial (i.e. metacentric) chromosomes preferentially pass to the egg is observed in chickens and humans, while in mice, monobrachial (i.e. acrocentric) chromosomes preferentially pass to the egg during oogenesis (de Villena and Sapienza 2001). Comparative approaches have shown that rapid switches in the directionality of this bias can explain patterns of chromosome number change across all mammals and replicated studies of four subclades (Chiroptera, Marsupials, Primates, and Cetartiodactyla). However, the analysis of the subclade Carnivora suggested that while rate variation is common in Carnivora, the female meiotic drive hypothesis failed to adequately explain the observed variation (Blackmon et al. 2019). Unlike many groups of mammals, Carnivora shows a less distinct bimodal distribution in karyotype morphologies, suggesting meiotic drive has been a weaker force in the evolution of karyotypes across this clade. The findings from this previous study suggest that other factors must explain variation in rates of chromosome number evolution in Carnivora. One possible explanation is variation in effective population size, which could allow mildly deleterious chromosome number mutations to be fixed in small populations via drift. Social structure in mammals has also been suggested as a trait that could produce small effective population sizes and may promote high rates of karyotype evolution through inbreeding and drift (Wilson et al. 1975; Bush et al. 1977). However, these studies lacked modern comparative methods that could account for phylogeny and apply probabilistic models of trait evolution.

To address our gaps in understanding the dynamics of Carnivora chromosome number evolution, we estimated rates of fusions and fissions while accounting for range size, which we use as a proxy for effective population size. While the relationship between effective population size and range size can be impacted by a variety of factors (e.g. social structure, population density, range fragmentation), we use range size as a proxy for effective population size as it is available for most species of Carnivora and should broadly be correlated with effective population size. We hypothesized that fusions and fissions are mildly deleterious or underdominant (Wodsedalek 1916; Ratomponirina 1988; Britton-Davidian et al. 1990; Pennell et al. 2015) and that lineages with small range sizes should exhibit higher rates of fusions and fissions since deleterious or underdominant mutations could more easily fix due to drift in these lineages. In this study, we find that the rates of fusions and fissions are elevated in taxa with small range sizes relative to those with large range sizes. Based on these findings, we conclude that 1) naturally occurring structural mutations that change chromosome number are underdominant or mildly deleterious, and 2) when population sizes are small, structural rearrangements may play an important role in speciation and reduction in gene flow among populations.

## Methods

### Data collection

We collected multiple data types for this study—chromosome number, range size, and phylogenies. First, we collected chromosome numbers from existing karyotypic compilations (de Villena and Sapienza 2001; Tree of Sex Consortium 2014; Blackmon et al. 2019; O’Brien et al. 2020). All karyotype data are available at www.karyotype.org. We define chromosome number as the haploid chromosome count in females. We use the female haploid chromosome number for ease as females produce haploid gametes with an equal number of chromosomes while males in species with complex XY sex chromosome systems (e.g. XXY or XYY) produce haploid gametes with a differing number of chromosomes. Next, we collected decimal coordinate data from the Global Biodiversity Information Facility (GBIF) for all available Carnivora species (Carnivora: GBIF Occurrence Download 2020). Using the gathered longitudinal and latitudinal data and the GeoRange library in R, we estimated the range size (in km^2^) for each Carnivora species (Boyle 2017). To ensure our method of range size estimation was appropriate, we compared our estimated range size calculations for 18 species to previously published data. Finally, we gathered species-level phylogenies from a previous study (Blackmon et al. 2019). The dataset of trees contained 100 trees from a posterior distribution using the 10k trees website and was used for all downstream comparative analyses (Arnold et al. 2010).

### Comparative analyses

We fit a model of chromosome number evolution to each tree from the posterior distribution. Our model contains two mechanisms for changes in chromosome number: rate of chromosome number decrease (fusion δ) and rate of chromosome number increase (fission γ). This model can also accommodate whole-genome duplication (polyploidy ρ); however, there is no support for polyploidy in Carnivora, so we used a constrained model that sets the rate of polyploidy to zero. For this model, each rate (fission and fusion) is estimated separately for lineages within each binary state. As range size is inherently a continuous trait, range size was discretized based on the median of all included species to assign species to either small or large range size, a requirement of the method being used. Additionally, two other parameters describe transitions between small and large range sizes (q_SL_ and q_LS_). This model was built using the R package chromePlus (Blackmon et al. 2019, 2023) and was fit using a Bayesian approach in the R package diversitree (FitzJohn 2012). All analyses were completed in R version 4.0.3 (Team and Others 2013), and scripts for all analyses and figures are available in a GitHub repository (https://github.com/coleoguy/carnivores).

Our Markov chain Monte Carlo (MCMC) was run for 500 generations on each of the 100 trees in the posterior distribution. Each MCMC run was initialized with parameter values drawn from a uniform distribution from 0 to 10 and an exponential prior with a shape parameter of 2 (favoring biologically realistic rates). Preliminary analyses were performed to estimate the tuning parameter for diversitree’s MCMC function (FitzJohn 2012). While running our MCMC models, we found most datasets converged quickly. However, a small subset of datasets explored areas of parameter space with relatively flat likelihood surfaces or remained “stuck” in an area of parameter space that was a local, though not global, peak in likelihood. Therefore, we ran the model with six replicates on each tree from our posterior distribution. We evaluated the six replicates by comparing the posterior probability of the final generation of the MCMC. We retained the MCMC run for each tree with the highest final posterior probability. From this final set of 100 MCMC runs, we removed the initial 450 generations as burnin and combined all post-burnin samples to form our final posterior distribution of rate estimates that account for phylogenetic uncertainty.

For purposes of model fitting, rates were inferred with trees transformed to unit length. All reported rates have been back transformed into units of millions of years (MY). As is customary for Markov models, the reported rates are Lamba parameters for exponential distributions that describe the expected waiting time for a transition. Because the central question driving our work is whether species with small effective population sizes have faster rates of karyotypic evolution than species with large effective population sizes, the results for our model are reported as a mean rate difference statistic, Δ*R*_*x*_, where the subscript *x* indicates the rate parameter. For example, with the rate of fusions (δ) for each post-burin sample, we calculated Δ*R*_δ_ as


$$\begin{array}{l} \Delta R_{\delta }=\delta_{small\;range\;size}-\delta_{large\;range\;size} \end{array}$$

The Δ*R* values for fusion and fission were plotted using ChromePlus’ plot function (Blackmon et al. 2019). In addition to estimating the magnitude of this statistic as a mean rate difference statistic, we also evaluated the 95% credible interval (CI) of Δ*R*_*x*_ (i.e. the 95% highest posterior density) calculated using Coda’s HPDInterval function (Plummer et al. 2006). If the entire 95% CI is positive, we interpret it as strong support for higher rates of chromosome number evolution in species with small range sizes. If the entire 95% CI is negative, we interpret it as strong support for higher rates of chromosome number evolution in species with large range sizes. Otherwise, we conclude there is little support for a significant rate difference in species with small and large range sizes.

### Tip rate reconstruction

To test whether one or a handful of species is driving our overall result, we developed a metric, “tip rates,” that can be calculated with the R function GetTipRates in the R package evobiR (Jonika et al. 2023). We define tip rate as the difference between the extant species’ chromosome number and the most probable chromosome number of the immediate ancestor of a given tip divided by the branch length between the immediate ancestor and the tip. Marginal ancestral state estimates were calculated using the asr_mk_model in the R package castor and the rate estimates from the above analyses (Louca and Doebeli 2018). Effectively, this approach allows us to check whether some species with “outlier” chromosome numbers might be driving high rate estimates.

### Rate heterogeneity analysis

We design and implement a novel method to quantify heterogeneity in rates of discrete character evolution across phylogenies. Our approach allows all branches in the phylogeny to be put into 21 rate bins. These bins are effectively scalars that range from 0.5 to 2 in equally sized increments. We perform a preorder traversal of the phylogeny, assuming the root is in a rate category of one. As each branch is visited, the branch is scaled by the rate bin of the parent branch as well as the bins directly above and below the parental bin and is assigned to the rate bin that maximizes the likelihood (effectively ensuring a degree of autocorrelation in rates across the phylogeny and avoiding overfitting). We performed this process for each tree in our posterior distribution using the mean rate matrix from our MCMC. Log likelihoods were estimated using the fitMk function in the R package phytools (Revell 2012). The proportion of edges experiencing elevated and depressed rates of change in each clade was averaged across our posterior distribution of trees. The rate values estimated for branches defining families with at least five taxa were pooled for downstream analyses.

### Chromosome number simulation and binary trait analysis

To test model adequacy and to see how well our binary trait captures heterogeneity in rates of chromosome number evolution, we used the simChrom function in the R package chromePlus (Blackmon et al. 2019). This function allowed us to create simulated chromosome number datasets using our posterior tree distribution. Parameters were sampled for the 100 simulations from the post-burn-in portion of the MCMC run on each tree. In all simulations, we set the root state to a haploid number of 19 (thought to be ancestral for Carnivora) (Nash et al. 2008). Finally, the hyperstate at the tree’s root was randomly sampled each time with a probability of 0.5 for either small or large range sizes. Chromosome numbers were simulated for each of the 100 trees in the posterior distribution.

It has become increasingly clear that many comparative methods have inherent weaknesses driven by heterogeneity in signal across phylogenies (Lotterhos et al. 2022). One of the most compelling examples of this is the elevated false-positive rate documented for the BiSSE method (Rabosky and Goldberg 2015). To test for the possibility of false positives under the chromePlus model, we simulated 100 neutral binary traits. Each of the 100 trees from our posterior distribution was used to simulate a single neutral binary trait. Each of these neutral traits was then analyzed on each of the 100 trees in our posterior distribution under an Mk-2 model using the R package diversitree just as the empirical data was. This simulated trait mimics the evolutionary dynamics of range size but clearly cannot impact inferred rates of chromosome evolution. We used a transition rate of two to mimic the transition rate we estimated for range size in our empirical data. Each simulation was started by randomly choosing a starting state and generating new observed states for the binary character. We then paired these neutral characters with our empirical phylogenies and chromosome numbers and repeated our analysis as described above. This approach will effectively allow us to measure our study’s expected false-positive rate. Scripts for all analyses are available in a GitHub repository (https://github.com/coleoguy/carnivores).

## Results

### Dataset collection and discretization of binary trait

**Fig. 1.**
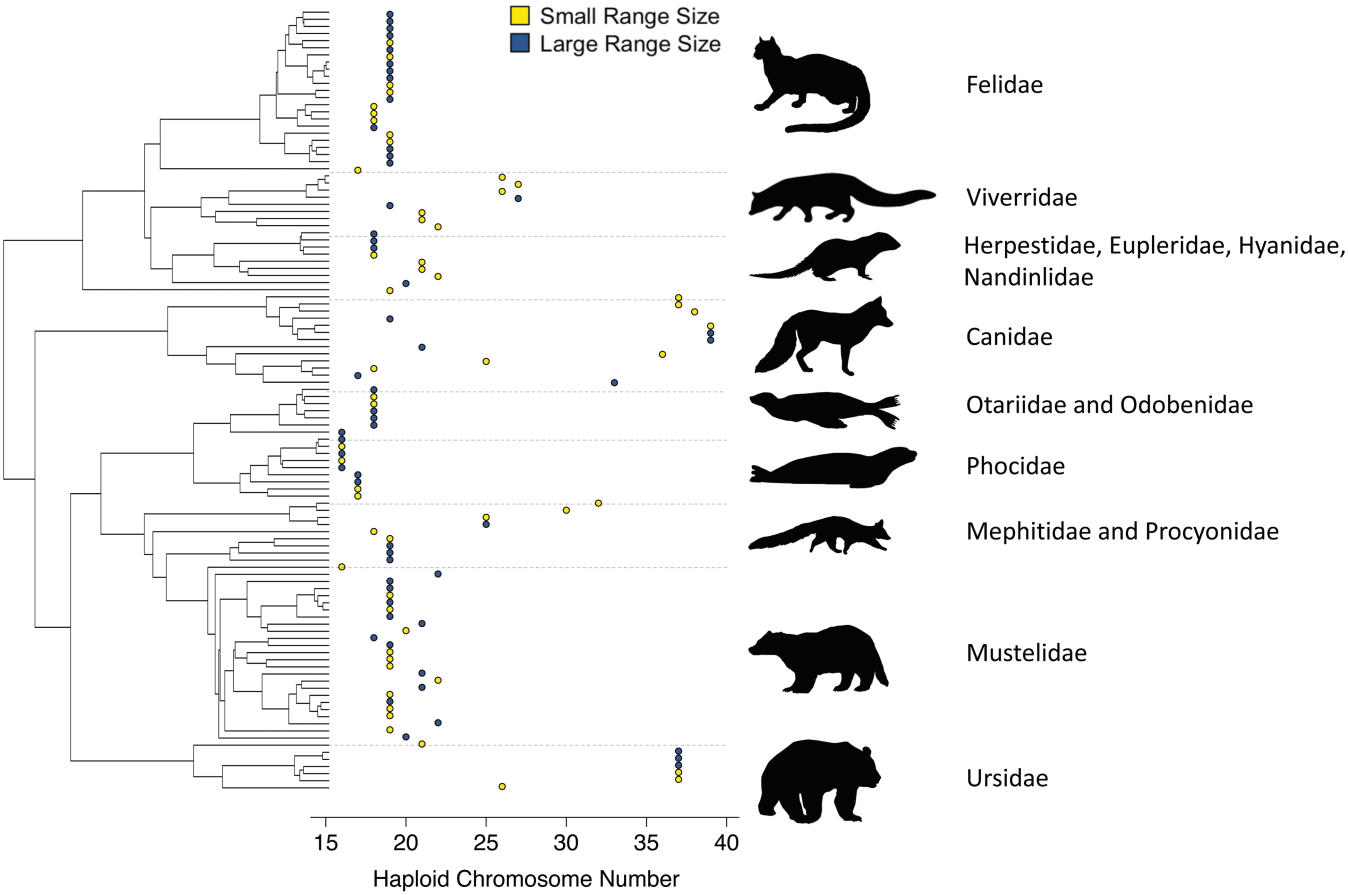
Phylogeny with range size and chromosome number. The outlined circles to the right of the phylogeny are colored to represent range size, with each circle indicating a species on the phylogeny. The yellow circles represent species with small range sizes, and the blue circles represent species with large range sizes. The *x*-axis indicates the haploid chromosome number for each species.

Our mammalian chromosome number dataset has 1,440 species comprising 29 orders, 136 families, and 604 genera. Within this mammalian chromosome number dataset, we have 168 Carnivora species comprising 92 genera and 14 families. In the Carnivora chromosome number dataset, the minimum female haploid chromosome number is 15, and the maximum haploid chromosome number is 39. From GBIF occurrence records, we obtained 1,457,081 observations for 1,023 Carnivora species. We subset this dataset to 1,030,610 observations for the 110 species in our Carnivora phylogeny. Our final range size dataset’s median species range size was approximately 29 million km^2^. There was a positive correlation between our estimated range size and published range size data, Pearson’s *r* = 0.61, *n* = 18, and this relationship is significant (*P* = 0.007) (Supplementary Table 1). For comparative analyses, we discretized range size based on the median value in the dataset, where observations greater than or equal to the median range size were classified as large range size, and observations less than the median range size were classified as small range size. Lastly, we used Carnivora species-level phylogenies from a previous study that included 502 tips (Blackmon et al. 2019). Our trait datasets and phylogeny overlapped 110 species that were used in all downstream analyses (Fig. 1).

### Rates of chromosome number evolution

**Fig. 2.**
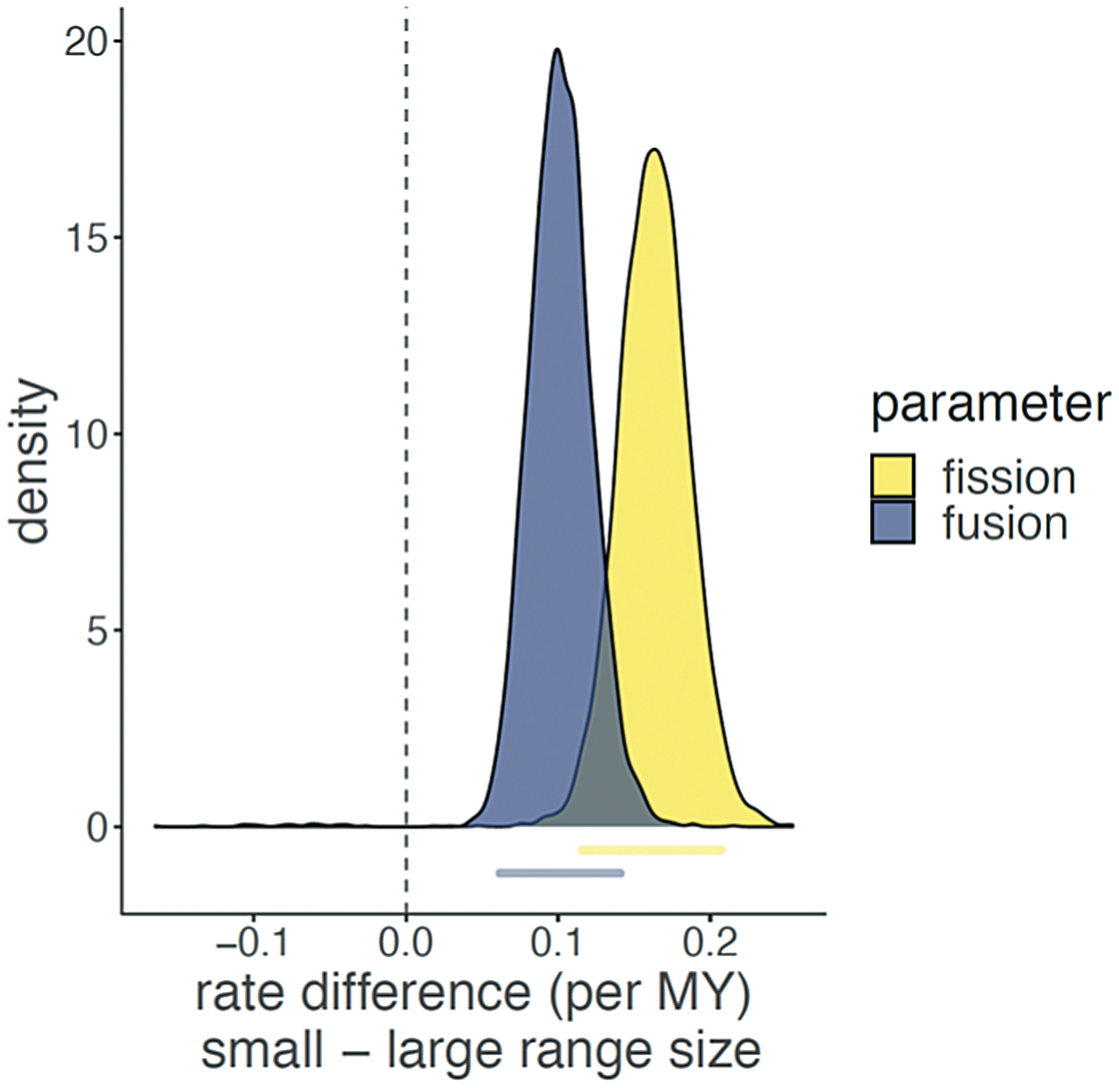
Rates of chromosome number evolution. Each curve represents the posterior distribution of the rate difference (per MY), where the rate difference can be either fission or fusion, indicated by the color of the fill (yellow and blue, respectively). Positive values indicate higher rates in lineages with small range size, while negative values indicate higher rates in lineages with large range size. Below each curve is a bar representing each statistic’s 95% CI. Under this model, the CI of both parameters, fission and fusion, are positive, indicating that species with small range sizes are associated with elevated rates of chromosome number evolution.

Because there is no evidence for polyploidy within Carnivora, we explored a basic model for chromosome number evolution that included only fusion and fission events. Each rate was estimated in species with small and large range sizes, allowing for transitions between small and large range sizes. This rate estimation was repeated for each of the 100 trees from our posterior distribution. We found a Δ*R*_fusion_ of 0.101 (CI 0.062 to 0.141) and a Δ*R*_fission_ of 0.163 (CI 0.116 to 0.207) (Fig. 2).

### Tip rate reconstruction

We calculated tip rates to determine whether one or a few taxa with extreme chromosome numbers may be driving our rate estimates. The minimum tip rate is 0, observed in 80 out of 110 species (Supplementary Fig. 1). The mean tip rate in species with small range sizes is 0.25, and the mean tip rate in species with large range sizes is 0.17. The maximum tip rate estimated was 5.15 for *Canis lupus* and *Canis latrans*, two species with 39 chromosomes classified as large and small range size, respectively. In our phylogenies, *C. latrans* and *C. lupus* are sisters to *Lycaon pictus* and *Cuon alpinus*. *Lycaon pictus* has a haploid chromosome number of 19, while *C. alpinus* has a haploid chromosome number of 39, and both are classified as having large range sizes. The fact that a species with a large range size and a small range size both have elevated tip rates suggests that no single species is driving our inferences in the primary analysis. Across all tip rates, we find a pattern where only 13 of the 55 large-range-size species have non-zero tip rates, while 17 of the 55 small-range-size species have non-zero tip rates. This characteristic of the data, where more species with small range sizes have a larger number of non-zero tip rates, is concordant with the patterns we see in our primary analysis. Furthermore, 30 of the 110 species show elevated tip rates, suggesting that the signal for elevated rates is present across many taxa.

### Rate heterogeneity analysis

**Fig. 3.**
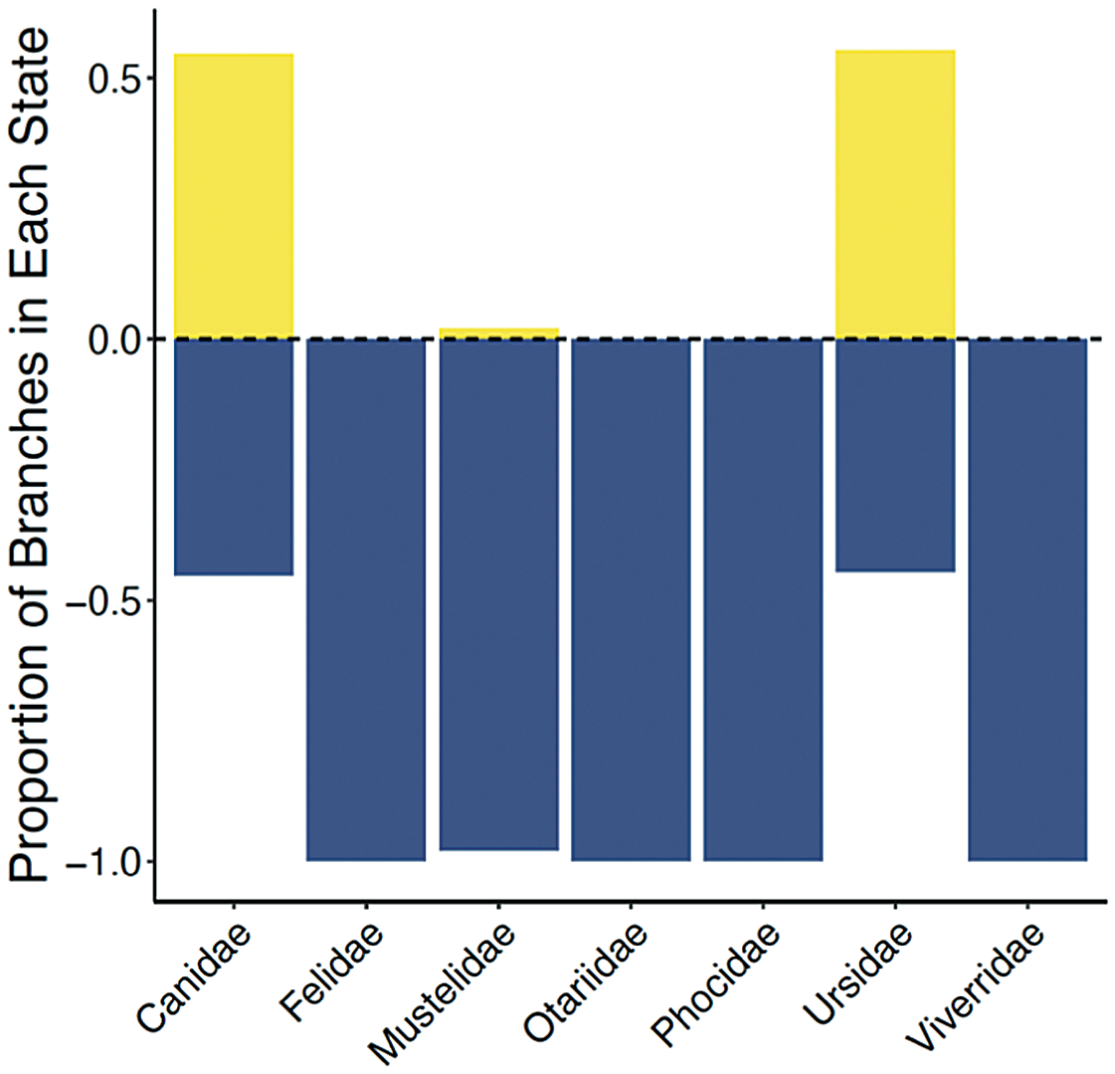
Scaled rates of chromosome number evolution. The horizontal axis represents each of the seven families that were included in the rate heterogeneity analysis. The vertical axis represents the proportion of branches in either a depressed (below zero) or elevated (above zero) state for their rates of chromosome number evolution.

We evaluated rate heterogeneity across seven families with at least five species in our dataset (Canidae, Felidae, Mustelidae, Otariidae, Phocidae, Ursidae, and Viverridae; Fig. 1) (Fig. 3). The minimum family mean scaled rate across our posterior distribution of trees was 0.63 in Mustelidae, and the maximum family mean scaled rate across our posterior distribution of trees was 0.98 in Ursidae and Canidae. The high mean scaled rate in Canidae further supports the increased tip rates reconstructed in Canidae above. Additionally, across all seven families, we find a pattern where Canidae and Urisdae include equal proportions of the clade (i.e. branches in the clade) experiencing depressed and elevated rates of chromosome evolution while the other five families are mostly experiencing depressed rates of chromosome number evolution (Fig. 3).

### Chromosome number simulation and binary trait analysis

**Fig. 4.**
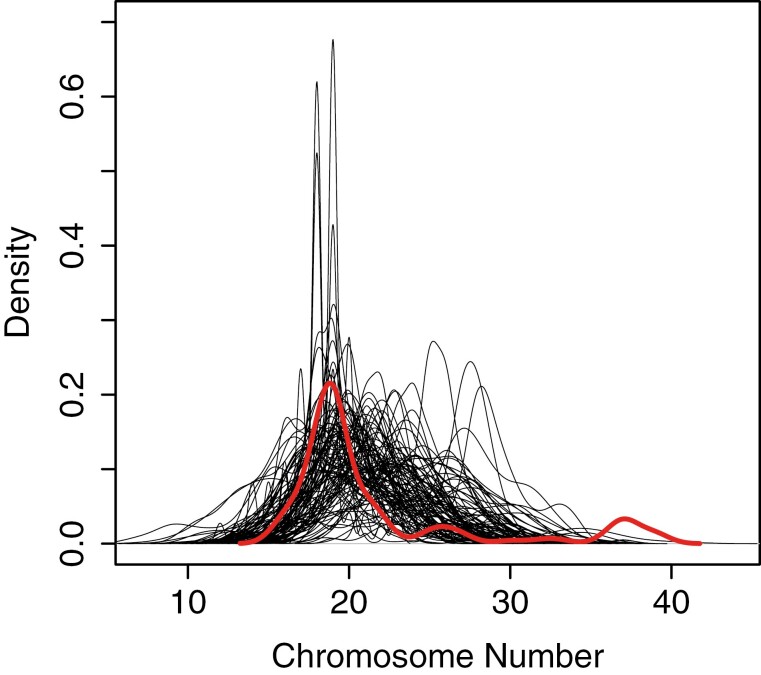
Variation in Carnivora chromosome number. Gray lines represent the distribution of chromosome numbers across simulated datasets. The red line indicates the distribution of chromosome numbers in the empirical dataset.

We compared empirical and simulated data by observing the variance across each of the simulated data sets compared to the empirical dataset. The variance in the empirical dataset was 39.8, while the range of variance in the simulated chromosome number datasets was 3.1 to 33.4, with an average of 10.2. While these variance values indicate differences between the simulated and empirical datasets, the range of chromosome numbers across the two datasets is similar (Fig. 4). This can be further explained when we observe tip rates among each binary state, where we find lower variation in chromosome number within each binary state than between binary states. These results suggest that while our range size classification captures some aspects of the evolutionary dynamics of our trait, it fails to produce datasets that exhibit the full degree of variation in the empirical data.

In our analysis of neutral binary traits, we considered a trait as producing a false positive if the CI of the Δ*R* statistic does not overlap zero. We found that 22% of neutral traits were associated with elevated rates of fusion, and 33% were associated with elevated rates of fissions. However, we note that the magnitude of the Δ*R* statistics was typically smaller than observed with the empirical dataset; only 7% of neutral traits produced mean Δ*R* statistics as high as observed for the empirical analysis. This suggests that while this dataset is at risk of producing considerable false positives, few false positives have as strong of a signal for differential rates of evolution as the empirical data.

## Discussion/Conclusion

Our findings suggest that effective population size may be a key factor shaping chromosome number evolution in Carnivora. There is a striking difference between the rates of chromosome number evolution between lineages with large and small range sizes, suggesting range size may substantially impact the fate of mutations that change chromosome number. A previous study investigated meiotic drive as a primary force in the evolution of mammalian chromosome numbers (Blackmon et al. 2019). While the meiotic drive is an excellent explanation for the rapid evolution of chromosome number in some clades, Blackmon et al. (2019) found little support for the role of meiotic drive in shaping chromosome number evolution in Carnivora. Our results suggest that while meiotic drive may have a small impact on shaping chromosome number evolution, range size has a much larger impact in Carnivora.

Population size plays an important role in the efficacy of selection and may be an important cause of variation in rates of chromosome number evolution. Chromosomal rearrangements are often assumed deleterious or underdominant and are thought to be more likely to fix in populations with meiotic drive or low effective population size (Ratomponirina 1988; Britton-Davidian et al. 1990; Escudero et al. 2016). However, the assumption that chromosomal rearrangements are deleterious or underdominant has often been based on the fitness of hybrids. This approach is challenging since both genic divergence and structural changes have occurred in the lineages that are being hybridized, and both factors could contribute to reduced fitness. In contrast, the comparative approach that we have used allows us to focus only on the fitness effects of the mutations that have reached fixation in a lineage and led to extant variation in chromosome number among species. We find elevated rates of chromosome number evolution in lineages with small range sizes relative to large range sizes in Carnivora. These results suggest that mutations that change chromosome numbers are mildly deleterious or underdominant, and chromosome number mutations are more likely to fix in species with small effective population sizes due to drift than in species with large effective population sizes where selection is more efficient. Continuing to model the impact of population size on estimated rates of chromosome number evolution within clades using modern comparative methods will be critical in advancing our knowledge of the predictors of rates of chromosome number evolution.

An example within Canidae emphasizes the importance of population size in karyotype evolution and genome restructuring. The raccoon dog, *Nyctereutes procyonoides*, exhibits varying chromosome numbers where continental populations from Asia and Europe have higher chromosome numbers (2*n* = 54) than Japanese island populations (2*n* = 38) (Wada and Imai 1991; Wada et al. 1991; Nie et al. 2003; Chueca et al. 2021). Karyotypic and paleontological data of the raccoon dog support island population karyotypes as derived through an unknown fusion mechanism. This example among raccoon dog populations supports the hypothesis that lineages with a reduced effective population size experience rapid and extensive karyotype remodeling. Contrasting continental or island populations, we expect island populations to have lower effective population sizes, reinforcing the idea that we see elevated rates of karyotype evolution in species with low effective population sizes.

Canidae presents a unique clade where we see elevated rates of chromosome number evolution with the largest tip rate values in *C. lupus* and *C. latrans*, the gray wolf and coyote, respectively, (Supplementary Fig. 1). Additionally, we find that across all of Carnivora, virtually all branches binned into elevated rate categories are in Canidae and Ursidae. In both clades, almost half of all branches are in elevated categories (Fig. 3). Literature suggests the ancestral Carnivora karyotype is likely a diploid chromosome number of 2*n* = 38 (Dutrillaux and Couturier 1983; Nash et al. 2008). While many Carnivora families maintain highly conserved karyotypes, other families like Canidae and Ursidae do not (Wurster-Hill 1973; Wurster-Hill and Gray 1975; Wurster-Hill and Centerwall 1982; Dutrillaux and Couturier 1983). Additionally, Canidae has the highest diploid number in Carnivora (2*n* = 78) and one of the most rearranged karyotypes (Fig. 1; Yang et al. 1999, 2000). Our elevated tip rates are consistent with these documented examples of karyotypic remodeling in Canidae and Ursidae.

To our knowledge, little consideration has been given to the degree of signal heterogeneity in models of chromosome evolution (but see Shafir et al. 2023). However, our results suggest that the rates of transitions estimated in common approaches may often be a balance between large portions of phylogenies that suggest very low rates of chromosome number evolution and a handful of branches that support strikingly high rates. This is clearly the case in our Carnivora dataset, where two clades (Ursidae and Canidae; Fig. 3) contain a strong signal for higher rates of chromosome number evolution. This problem is not terribly different from the challenges identified in similar models like BiSSE (Rabosky and Goldberg 2015). To evaluate the degree to which this concentration of signal is driving our results, we completed three additional analyses.

First, we looked at the impact of another trait, body size. Using the same model from our range size analysis, we find higher rates of chromosome number evolution in Carnivora lineages with small body sizes (Supplementary Fig. 2). This result is likely caused by the fact that most Canidae are grouped with small body sizes where the signal for high rates is the strongest. While it may be expected that small body sizes are associated with larger effective population sizes, this relationship is complicated by a number of factors (Ozgul et al. 2010; Hilbers et al. 2017). Based on these results, we then performed an analysis of simulated neutral binary traits to determine a false positive rate for our primary analysis. Our simulation of 100 neutral traits led to false positive rates ranging from 22% to 33%. However, we also found that the magnitude of Δ*R* statistics for these neutral traits was less extreme in all but 7% of simulated traits. This suggests that the strength of signal concentration in our empirical dataset is strong but not so strong as to typically lead to Δ*R* statistics as extreme as were estimated using the empirical data. Finally, to further confirm that our result wasn’t being driven solely by the mapping of small range size onto Canidae, we repeated our primary analysis after pruning Canidae from our dataset. In this supplemental analysis, we still recover elevated rates of chromosome fission in lineages with small range size, however, not rates of chromosome fusion (Supplementary Fig. 3).

The degree to which concentrated signals of heterogeneity are responsible for positive findings in studies of this type is an area that should be actively pursued when using methods inferring differential rates of chromosome number evolution (Zenil-Ferguson et al. 2018; Blackmon et al. 2019). Newer methods that allow for agnostic searches for regimes of chromosome evolution (Shafir et al. 2023) or methods like ours that allow practitioners to query all branches of a tree for their impact on rates will be essential moving forward.

Changes in chromosome number can impact our understanding of divergence (Burt et al. 2009; Garagna et al. 2014), adaptation (Guerrero and Kirkpatrick 2014), and speciation (White 1978; Rieseberg 2001). Investigating traits that increase karyotype rearrangement and genome restructuring is essential to understanding diversity patterns across the tree of life. Despite this, we are only beginning to understand how chromosome number evolves. In this study, we have focused on the dynamics of chromosome number in Carnivora. However, recent studies have investigated the dynamics of chromosome number evolution in various clades encompassing extensive time spans on the tree of life (Blackmon and Demuth 2014; Ross et al. 2015; Blackmon et al. 2019; Ruckman et al. 2020; Sylvester et al. 2020). Before these studies, chromosome number was only studied on small clades in isolation (Rockman and Rowell 2002; De Oliveira et al. 2015; McCann et al. 2016). Studying chromosome numbers in isolation may present a challenge as variation in the inferred rates of chromosome number evolution is influenced by topologies and branch lengths inferred in each study. The recent development of multiple probabilistic models of chromosome number evolution allowing for associations between speciation or binary characters offers a way for us to study factors impacting rates of chromosome number evolution and, ultimately, genome diversity across the tree of life (Freyman and Höhna 2018; Zenil-Ferguson et al. 2018; Blackmon et al. 2019). Further study into large clades across the tree of life with these probabilistic models can help us untangle the importance of natural selection and genetic drift in the evolution of genome structure and its possible role in speciation.

## Supplementary Material

Supplementary material is available at *Journal of Heredity* Journal online.

esae025_suppl_Supplementary_Tables_1_Figures_1-3

## Data Availability

All data and scripts necessary to replicate the analyses and figures in this manuscript are available via a GitHub repository: https://github.com/coleoguy/carnivores.
