# Supplementary material for: Drift drives the evolution of chromosome number II: The impact of range size on genome evolution in Carnivora
Source: J Hered. 2024 May 7;115(5):524–31. doi: 10.1093/jhered/esae025 (PMC11334210; doi:10.1093/jhered/esae025)
Supplement: esae025_suppl_Supplementary_Tables_1_Figures_1-3 [file esae025_suppl_supplementary_tables_1_figures_1-3.docx]

**SUPPLEMENT**

**Supplementary Table 1. Range Size Estimates.** Range size estimates for our study and previously published range size estimates.

| Species | Our study’s range size estimate | Previous study’s range size estimate |
| --- | --- | --- |
| *Acinonyx jubatus* | 78195248 | 21775322 |
| *Canis lupus* | 296157519 | 22424810 |
| *Chrysocyon brachyurus* | 26118107 | 3420042 |
| *Crocuta crocuta* | 198529511 | 16838810 |
| *Eira barbara* | 34735889 | 1062902 |
| *Felis silvestris* | 280902548 | 4254032 |
| *Gulo gulo* | 50383153 | 2074878 |
| *Leopardus pardalis* | 36989257 | 2118876 |
| *Leptailurus serval* | 135633288 | 18747059 |
| *Lutra lutra* | 119983642 | 6875291 |
| *Lynx lynx* | 54009008 | 5174066 |
| *Mustela lutreola* | 33960248 | 3612346 |
| *Mustela nigripes* | 5521897 | 1997280 |
| *Panthera leo* | 196892011 | 19529625 |
| *Panthera pardus* | 199687819 | 24544214 |
| *Panthera tigris* | 72672201 | 8864559 |
| *Puma concolor* | 143074528 | 13417342 |
| *Ursus arctos* | 88309707 | 17634224 |


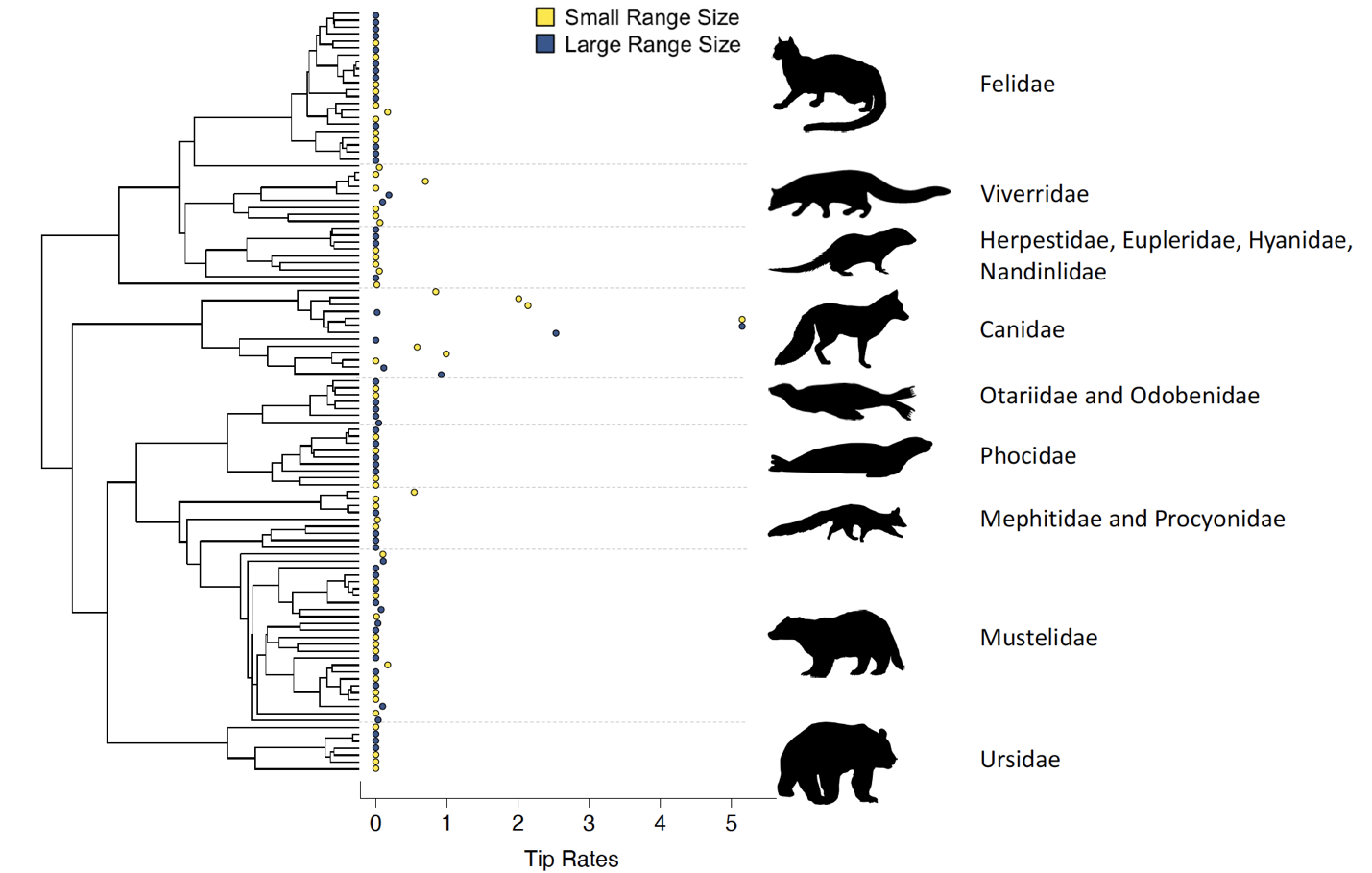


**Supplementary Figure 1: Tip rate reconstruction in Carnivora.** The distance of each bar across the x-axis represents the averaged posterior distribution of the tip rate for each species. Yellow dots represent species with small range sizes, and blue dots indicate species with large range sizes.


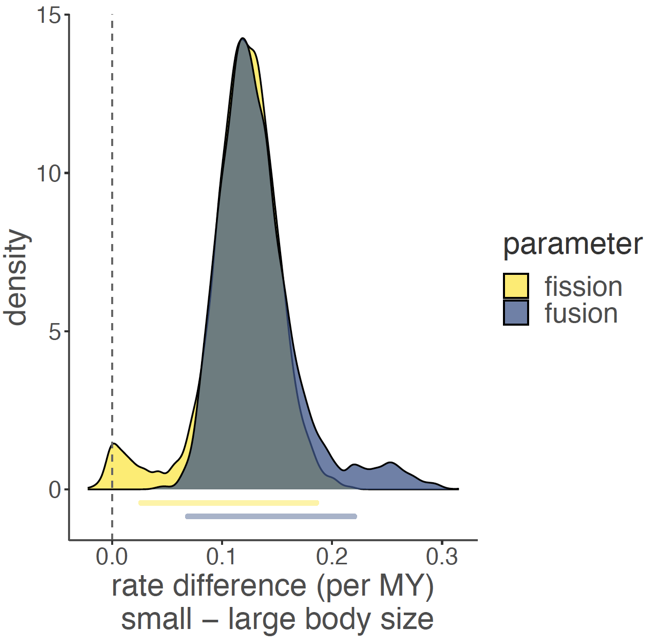


**Supplementary Figure 2. Rates of chromosome number evolution.** Each curve represents the posterior distribution of the rate difference (per MY), where the rate difference can be either fission or fusion, indicated by the color of the fill (yellow and blue, respectively). Positive values indicate higher rates in lineages with small body sizes, while negative values indicate higher rates in lineages with large body sizes. Below each curve is a bar representing each statistic's 95% CI. Under this model, the CI of both parameters, fission and fusion, are positive, indicating that species with small body sizes are associated with elevated rates of chromosome number evolution.


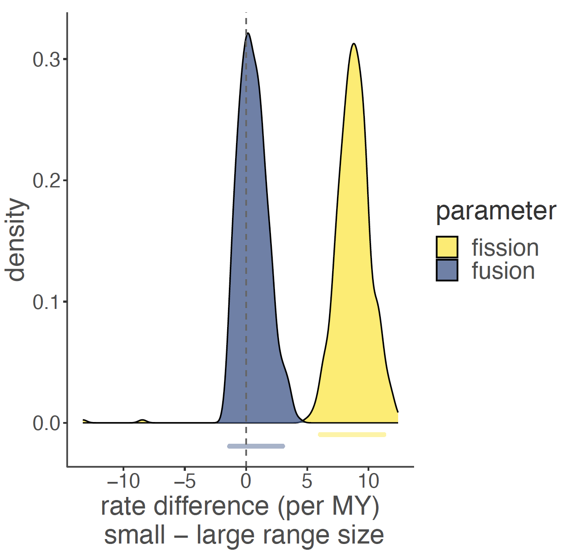


**Supplementary Figure 3. Rates of chromosome number evolution.** Each curve represents the posterior distribution of the rate difference (per MY), where the rate difference can be either fission or fusion, indicated by the color of the fill (yellow and blue, respectively). Positive values indicate higher rates in lineages with small range size, while negative values indicate higher rates in lineages with large range size. Below each curve is a bar representing each statistic's 95% CI. Under this model, the CI of fissions is positive, indicating that species with small range sizes are associated with elevated rates of fissions, while the CI of fusions overlaps zero, indicating that species with small or large range sizes are not associated with elevated rates of fusions.
